# Supplementary material for: Neuroprotective Effects of a Hydrogen Sulfide Donor in Streptozotocin-Induced Diabetic Rats
Source: Int J Mol Sci. 2023 Nov 23;24(23):16650. doi: 10.3390/ijms242316650 (PMC10706751; doi:10.3390/ijms242316650)
Supplement: Supplementary file 1 [file ijms-24-16650-s001.zip › ijms-2627156-supplementary.pdf]

## Supplemental Methods

*Neurobehavioral Sensory Tests*

1. Von Frey Filaments

The animals' response to non-nociceptive mechanical stimuli was evaluated by determining 50% mechanical threshold for responses to mechanical stimuli, which was applied to experimental animals' hind paws after the 2nd and 4th week of the experiment using Von Frey (vF) filaments (Ugo Basile SRL, Italy), as described previously [1,2]. A single filament was brushed across the skin to create a dynamic stimulus, and a single filament was applied statically three times to create a static stimulus. Using a complete set of these filaments with different bending forces, allodynia thresholds were evaluated. In addition to the paw withdrawal reflex, supraspinal indications included licking, running away, and head orientation change were taken as responses[3-8]. This test detects the frequency of responses to a series of stimuli. When a response is absent, the next stronger hair is used, but if a response is present, the next lighter hair is used.

2. Paw Pressure Test

The paw pressure test was used to measure response to nociceptive mechanical stimuli in animals by observing the reaction to gradually increasing pressure on a paw, a previously described [2,9,10]. If the rat started to exhibit the flight or struggle response, the nociception was deemed present. A cone-shaped pusher with a rounded tip, of the analgesic meter (Ugo Basile SRL, Italy) which did not hurt the rat, was used to apply the force on the rat's paw, which was placed on a small plinth. To start the mechanism, the operator depressed a pedal-switch by exerting force. When the rat struggled, it was the moment when the operator released the pedal. Then, the scale reading was taken. This test was done two times after the 2nd and 4th week of the experiment. Additional weights were provided to increase the force range. The equations used for left hind and right hind are (Left hind − Right hind/left hind * 100) and (Right hind − Left hind/Right hind * 100), respectively.

3. Hot Plate Analgesia Test

To measure response to thermal nociception, a hot plate test apparatus (Ugo Basile SRL, Italy) was used. As explained previously, the rat was placed on a pre-heated plate at 50 °C within the hot plate apparatus [2]. The time required for the rats to lick the paw was recorded manually and an average of three readings average was used. In a previous study, paw licking was used as a sign that the supraspinal processing of nociception was taking place [11]. A cut-off time of 30 s was established to minimize an animal's risk of sustaining tissue injury from prolonged exposure to the heated surface.

## Supplemental Results

*Effect of treatment of streptozotocin-diabetic rats with GYY4137 on neurobehavioral sensory tests*

*Von Frey test*

There was a significant difference in withdrawal threshold between the four groups during the week 2 and 4 (Figure S1). Diabetic animals developed mechanical allodynia i.e., there was a significant reduction in the mechanical threshold, in the diabetic group compared to the control group (P = 0.0012). Treatment with GYY4137 prevented the development of mechanical allodynia i.e., there was no significant difference between the GYY4137-treated diabetic group and the control group (P > 0.05). In addition, the withdrawal threshold of the GYY4137-treated diabetic group was significantly higher (P < 0.0001) in comparison to diabetic animals.

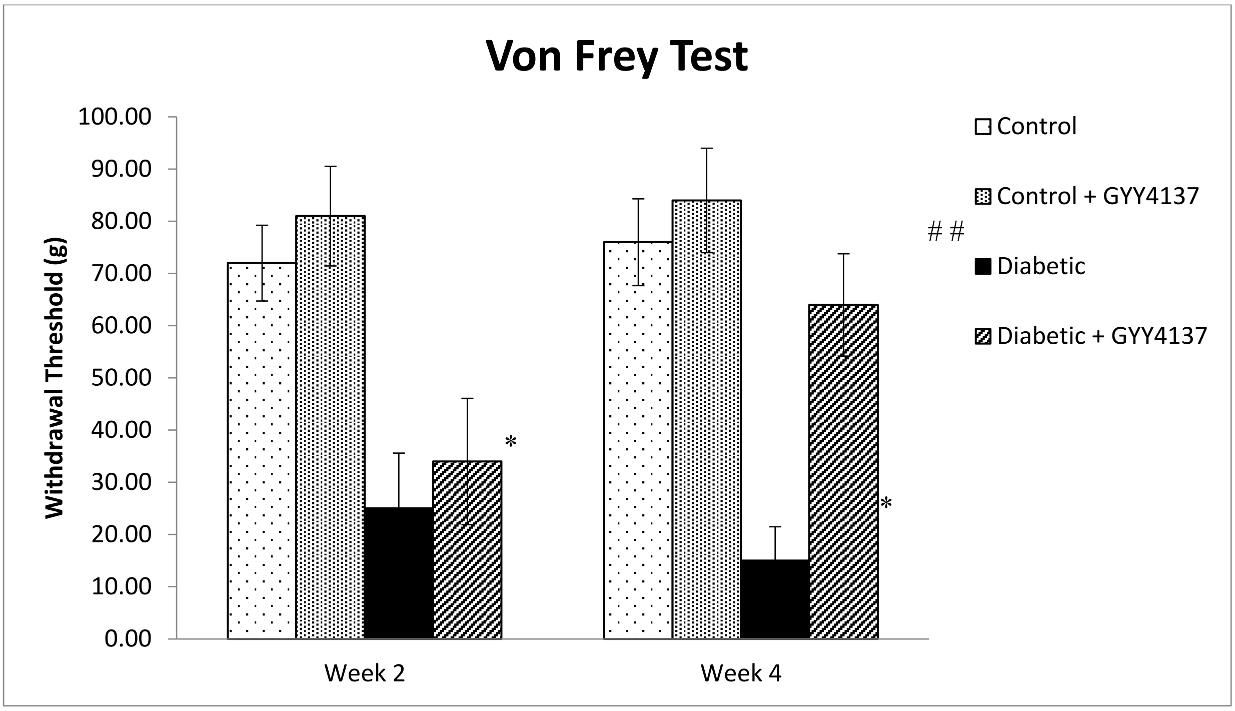


**Figure S1:** Response threshold force to the Von Frey test of each group throughout weeks two and four. (*P = 0.0012) Diabetic group vs. Control group at week 4. (##P < 0.0001) Diabetes + GYY4137 group vs. Diabetic group at week 4 (two-way ANOVA followed by Bonferroni post-test). Values are mean ± SD, n=10.

*Paw Pressure test*

There was a significant increase in paw withdrawal threshold to the force produced by the UGO Basile paw pressure instrument in both control rats i.e., not treated and treated with GYY4137, in the left hind limb between the 2nd and 4th-week interval (Figure S2 (A)). In contrast, there was a significant decrease in the paw withdrawal reflex threshold, the place or point of entering or beginning the pain, (P = 0.025) in diabetes-induced rats at week 2 in the left hind limb compared to control groups. Diabetic rats treated with GYY4137 significantly (P = 0.04)

improved with time compared to untreated diabetic rats. In addition, there was no significant difference between GYY4137-treated diabetic and control rats during week 2. The paw withdrawal reflex threshold in the left hind limb of diabetes-induced rats was substantially lower in the fourth week ($P < 0.001$) than in the second week compared to control groups. Also, during the fourth week, diabetic rats treated with GYY4137 displayed a robust significant difference compared to untreated diabetic rats ($P = 0.016$). In addition, GYY4137-treated diabetic rats did not show a significant difference compared to control rats during week 4 ($P = 0.066$). Likewise, the right hind limb showed a significant decrease in paw withdrawal threshold in the STZ-induced diabetic animals ($P = 0.004$) compared to the control groups and GYY4137-treated group (Figure S2 (B)). Furthermore, there was a significant improvement in the paw withdrawal threshold of GYY4137-treated diabetic rats compared to diabetic rats ($P = 0.003$). Moreover, there was no significant difference between GYY4137-treated diabetic rats and control rats during week 2. Also, during the fourth week, diabetic rats treated with GYY4137 displayed a significant difference from diabetic rats ($P < 0.001$). Also, during the fourth week ($P = 0.055$), diabetic rats treated with GYY4137 did not significantly differ from the control rats.

**(A)**

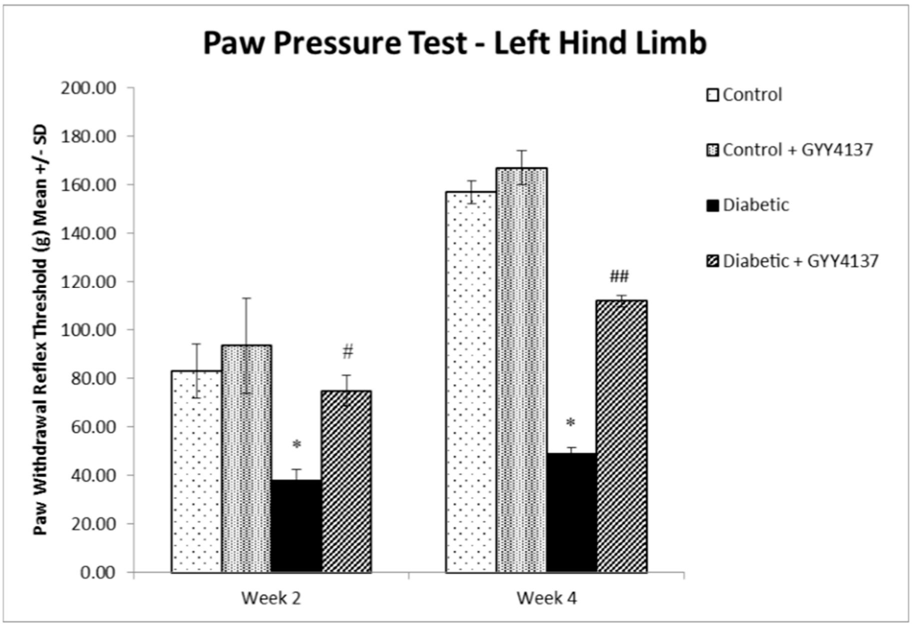


**(B)**

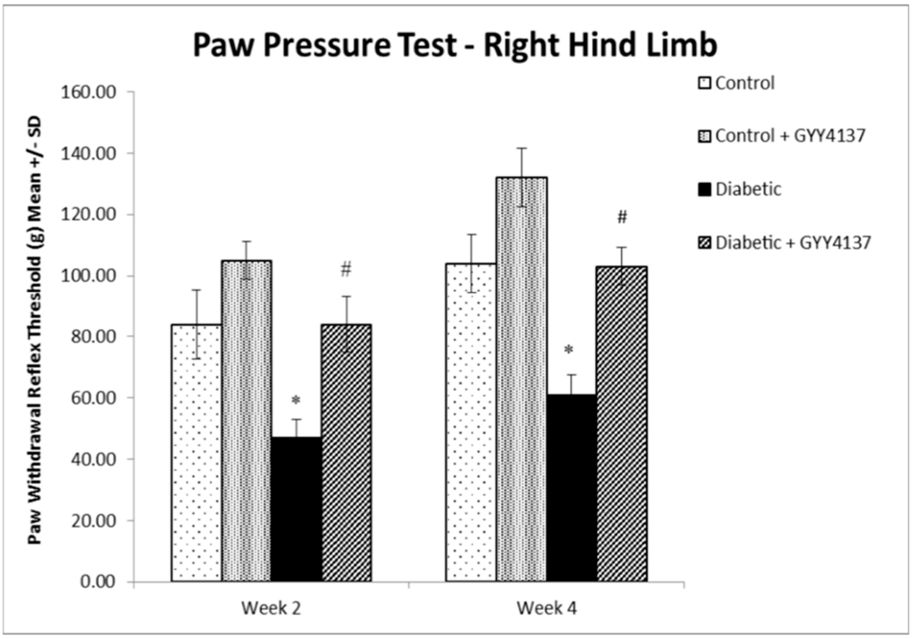


**Figure S2: (A)** All experimental groups showed paw withdrawal reflexes in response to pressure administered to the left hind limb at week 2 and week 4 intervals. At week 2, (*P = 0.025) Diabetic group vs. Control group. At week 2, (#P = 0.04) Diabetic + GYY4137 group vs. Diabetic group. (##P = 0.016) Diabetic + GYY4137 group vs. Diabetic group at week 4 (two-way ANOVA followed by Bonferroni post-test). **(B)** Results of the right hind limb paw pressure analysis for all experimental groups are displayed in a bar graph between weeks 2 and 4. (*P = 0.004) Diabetes group vs. Control groups at week 2. (#P = 0.003) at week 2, between the Diabetes + GYY4137 group and the Control group. At week 4 (*P = 0.055), Control groups vs. Diabetes + GYY4137 group (two-way ANOVA followed by Bonferroni post-test). Values are mean ± SD, n=10.

*2.2.3 Hot plate test*

Hot plate tests were applied for each rat at week 2 and week 4 (Figure S3). There was a significant increase in the level of withstanding heat when provoked by heat stimulus during week 2 in the diabetic rats (P = 0.027), but the GYY4137-treated diabetic rats did not show a significant difference compared to the control group during week 2. In addition, there was a significant decreasein the respnse time to heat nociception in GYY4137-treated diabetic rats compared to diabetic rats at week 2 and week 4 (P = 0.001).

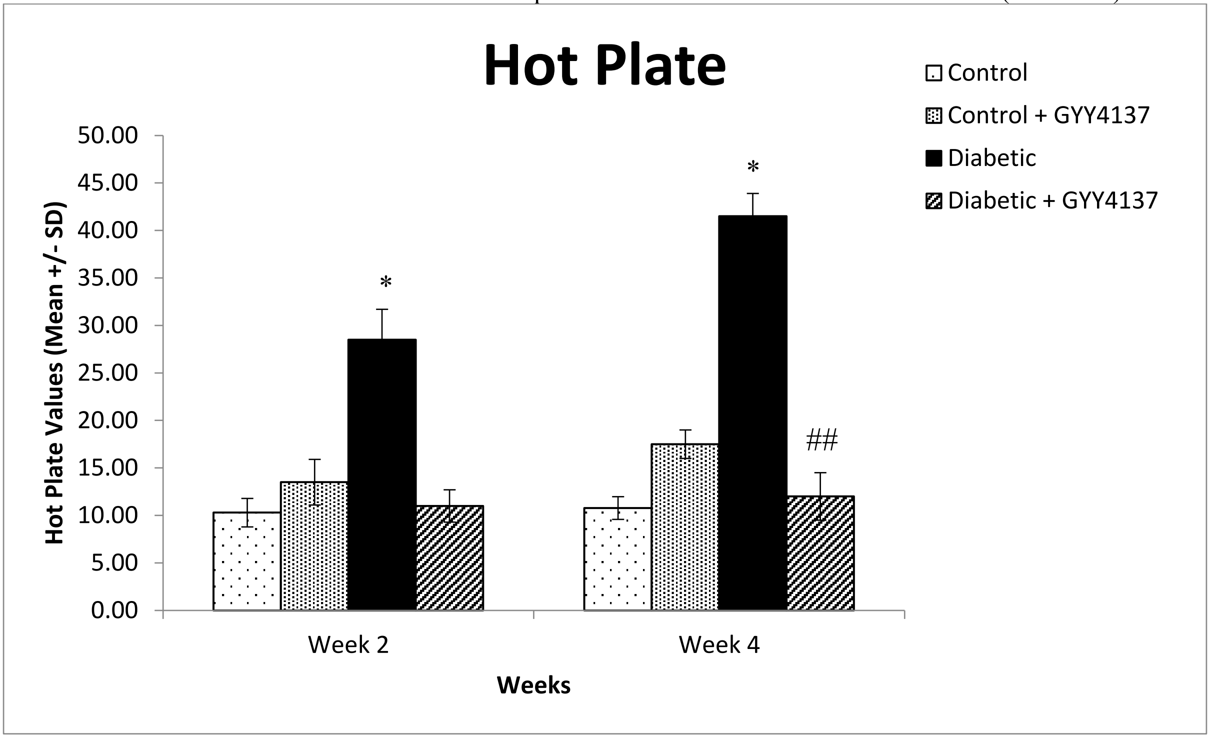


**Figure S3:** Reaction latency times to the hot plate test of all the experimental groups at week 2 and week 4 intervals. (*P = 0.027) Diabetic vs. Control, Control + GYY4137 and Diabetic + GYY4137 groups. (# #P < 0.001) Diabetic + GYY4137 vs. Diabetic group (two-way ANOVA followed by Bonferroni post-test). Values are mean ± SD, n=10.

**References**


.1 Renno, W.M.; Saleh, F.; Klepacek, I.; Al-Khaledi, G.; Ismael, H.; Asfar, S. Green tea pain modulating effect in sciatic nerve chronic constriction injury rat model. *Nutr Neurosci* **2006**, *9*, 41-47, doi:10.1080/10284150600576705.

.2 Shayea, A.M.F.; Mousa, A.M.A.; Renno, W.M.; Nadar, M.S.; Qabazard, B.; Yousif, M.H.M. Chronic Treatment With Hydrogen Sulfide Donor GYY4137 Mitigates Microglial and Astrocyte Activation in the Spinal Cord of Streptozotocin-Induced Diabetic Rats. *J Neuropathol Exp Neurol* **2020**, *7* ,1343-1320 ,9doi:10.1093/jnen/nlaa127.

.3 Chaplan, S.R.; Bach, F.W.; Pogrel, J.W.; Chung, J.M.; Yaksh, T.L. Quantitative assessment of tactile allodynia in the rat paw. *J Neurosci Methods* **1994**, *53*, 55-63, doi:10.1016/0165-0270(94)90144-9.

.4 Yoon, C.; Wook, Y.Y.; Sik, N.H.; Ho, K.S.; Mo, C.J. Behavioral signs of ongoing pain and cold allodynia in a rat model of neuropathic pain. *Pain* **1994**, *59*, 369-376, doi:10.1016/0304-3959(94)90023-x.

.5 Ho Kim, S.; Mo Chung, J. An experimental model for peripheral neuropathy produced by segmental spinal nerve ligation in the rat. *Pain* **1992**, *50*, 355-363, doi:10.1016/0304-3959(92)90041-9.
.6 Christensen, M.D.; Hulsebosch, C.E. Chronic central pain after spinal cord injury. *J Neurotrauma* **1997**, *14*, 517-537, doi:10.1089/neu.1997.14.517.
.7 Hao, X.J.; Xu, J.X.; Aldskogius, H.; Seiger, Å.; Wiesenfeld-Hallin, Z. Allodynia-like effects in rat after ischaemic spinal cord injury photochemically induced by laser irradiation. *Pain* **1991**, *45*, 175-185, doi:10.1016/0304-3959(91)90186-2.
.8 Yoon, Y.W.; Dong, H.; Arends, J.J.; Jacquin, M.F. Mechanical and cold allodynia in a rat spinal cord contusion model. *Somatosens Mot Res* **2004**, *21*, 25-31, doi:10.1080/0899022042000201272.
.9 Lal, H.; Fielding, S. Psychopharmacology of clonidine: an introduction. *Prog Clin Biol Res* **1981**, *71*, 1-3.
.10 Blivis, D.; Haspel, G.; Mannes, P.Z.; O'Donovan, M.J.; Iadarola, M.J. Identification of a novel spinal nociceptive-motor gate control for Aδ pain stimuli in rats. *Elife* **2017**, *6*, e23584, doi:10.7554/elife.2.3584
.11 Christensen, M.D.; Everhart, A.W.; Pickelman, J.T.; Hulsebosch, C.E. Mechanical and thermal allodynia in chronic central pain following spinal cord injury. *Pain* **1996**, *68*, 97-107, doi:10.1016/s0304-3959(96)03224-1.
